# Rapid processing of chemosensor transients in a neuromorphic implementation of the insect macroglomerular complex

**DOI:** 10.3389/fnins.2013.00119

**Published:** 2013-07-12

**Authors:** Timothy C. Pearce, Salah Karout, Zoltán Rácz, Alberto Capurro, Julian W. Gardner, Marina Cole

**Affiliations:** ^1^Centre for Bioengineering, Department of Engineering, University of LeicesterLeicester, East Midlands, UK; ^2^Microsensors and Bioelectronics Laboratory, School of Engineering, University of WarwickCoventry, West Midlands, UK

**Keywords:** machine olfaction, ratiometric processing, neuromorphic model, pheromone processing, infochemical communication, transient processing

## Abstract

We present a biologically-constrained neuromorphic spiking model of the insect antennal lobe macroglomerular complex that encodes concentration ratios of chemical components existing within a blend, implemented using a set of programmable logic neuronal modeling cores. Depending upon the level of inhibition and symmetry in its inhibitory connections, the model exhibits two dynamical regimes: fixed point attractor (winner-takes-all type), and limit cycle attractor (winnerless competition type) dynamics. We show that, when driven by chemosensor input in real-time, the dynamical trajectories of the model's projection neuron population activity accurately encode the concentration ratios of binary odor mixtures in both dynamical regimes. By deploying spike timing-dependent plasticity in a subset of the synapses in the model, we demonstrate that a Hebbian-like associative learning rule is able to organize weights into a stable configuration after exposure to a randomized training set comprising a variety of input ratios. Examining the resulting local interneuron weights in the model shows that each inhibitory neuron competes to represent possible ratios across the population, forming a ratiometric representation via mutual inhibition. After training the resulting dynamical trajectories of the projection neuron population activity show amplification and better separation in their response to inputs of different ratios. Finally, we demonstrate that by using limit cycle attractor dynamics, it is possible to recover and classify blend ratio information from the early transient phases of chemosensor responses in real-time more rapidly and accurately compared to a nearest-neighbor classifier applied to the normalized chemosensor data. Our results demonstrate the potential of biologically-constrained neuromorphic spiking models in achieving rapid and efficient classification of early phase chemosensor array transients with execution times well beyond biological timescales.

## Introduction

Animal survival depends upon effective communication between individuals of the same and different species. In insects, olfactory cues in the form of pheromones have long been established to play a crucial role in such conspecific and interspecific signaling (Wyatt, 2003). Through evolutionary pressure many insect species have become exquisitely sensitive to specific pheromones, achieved via highly specialized detection pathways of the olfactory system, selectively adapted for robust and rapid chemoreception. The male moth for instance, for which pheromones mediate mate location, has been found to be responsive to minute quantities of the female-produced pheromone blend hitting the antenna, as quantified through both behavioral and physiological responses (Kaissling, 1987; Angioy et al., 2003). Since female moths produce pheromone blend amounts of the order of nanograms per hour through endocrine production and exocrine release, the corresponding level of sensitivity in the male must be extreme to support olfactory guided localization over relatively large distances during flight (Tillman et al., 1999). Considering only the most likely molecular modifications of long-chain hydrocarbons used for signaling in moths, over 100,000 different pheromones could potentially exist, yet each subspecies responds to a small subset of individual compounds in precise ratios, demonstrating that this extreme level of sensitivity is also combined with high specificity (Martin and Hildebrand, 2010).

In a remarkable example of an olfactory guided behavior, male moths navigate pheromone-laden plumes comprising fine filamentous structures over large distances in order to locate calling females, necessitating pheromone signals to be detected both rapidly and robustly (Vickers and Baker, 1994; Vickers and Christensen, 2003). The signal produced by the female is made yet more specific and informative because the pheromones are generally produced in blends, involving more than one molecular cue. The chemical composition, number of components and ratio of concentrations of these blends appears to be important to the pheromone detection task, since different species often use similar chemicals in a different context, and interfering with blend composition can block appropriate behavioral responses (Mustaparta, 1996). Using ratios in this way confers the important advantage of concentration invariance when communicating through highly intermittent plume structures. Thus, insect-based pheromone signaling provides an ideal model for information-rich concentration invariant communication that could provide an alternative to ubiquitous electronic communication.

Which features of the insect olfactory pathway are functionally relevant to achieving such sensitive, selective, rapid and robust chemical communication? An important clue is provided by the fact that in many insect species the olfactory pathway is sexually dimorphic—in particular males have pheromone olfactory receptors coupled with specialized brain areas for processing signals from these receptors at the first site of neural processing, the antennal lobe (AL) (Christensen et al., 1989; Anton and Homberg, 1999; King et al., 2000; Rospars and Hildebrand, 2000). Males have large glomeruli contained within this area, the macroglomerular complex (MGC) that are responsive exclusively to sex-pheromones. Thus, the MGC can be considered to be a key pheromone processing subsystem of the insect brain, largely separate to the rest of the olfactory system, containing two types of neurons: local interneurons (LNs) and projection neurons (PNs). LNs and PNs within the MGC may also be sexually dimorphic (Homberg et al., 1988, 1989), further suggesting a specialized role in female produced pheromone processing in males. Pheromone specific olfactory receptor neuron (ORN) axons on the periphery project exclusively to the MGC region, with those ORNs expressing identical pheromone sensitive olfactory receptors converging on a single macroglomerulus, thought to enhance signal-to-noise ratio to optimize pheromone sensitivity (Pearce et al., 2001).

Within the MGC region of the AL it is clear that a considerable degree of processing takes place. ORNs synapse directly onto both LNs and PNs, as an excitatory drive of the pheromone processing pathway. LNS connect more or less to all glomeruli, allowing lateral interactions within the structure as a whole. The final output of this MGC processing is delivered to higher brain areas via the PN population. These neurons interconnect predominantly within a single glomerulus (uniglomerular PNs) and lead the signal to higher brain areas including the mushroom bodies and the lateral brain. Importantly, some of these AL neurons in the male moth have been shown to exhibit so-called blend specificity that may have special behavioral importance in terms of pheromone blend ratiometric detection (Wu et al., 1996; Heinbockel et al., 2004). Blend encoding neurons have been identified that only respond to an olfactory stimulus containing all behaviorally relevant pheromone components produced by the conspecific female in the correct ratio between these components (Hansson et al., 1994; Hansson and Anton, 2000). Consequently we see evidence for a type of ratiometric processing of pheromone blend information, in which networks of neurons appear to be performing a computation to identify specific blends of individual molecular ligands occurring in appropriate ratios.

What can we learn from these neural structures to build more capable chemical sensing instrumentation? Using ratios in this way provides a robust encoding mechanism when communicating that we have exploited in the first example of an infochemical communication system based upon both moth biosynthesis and ratiometric chemoreception (Cole et al., 2009a,b; Rácz et al., 2011, 2013). This technological objective together with understanding the underlying neural mechanisms for ratiometric decoding, motivate this study as the insect MGC system provides an ideal model system to investigate the close relationships between external input stimuli and internal brain dynamics.

In this paper we consider the first station of pheromone processing in the insect brain, the MGC, in terms of achieving rapid and robust ratiometric processing of chemosensory information. Through building a biologically-constrained model of the MGC structure and deploying in neuromorphic hardware, we test the ratiometric processing capabilities of the system directly, enabling us to investigate its ability for rapid and robust classification of ratiometric input. We specifically focus on two problems: can associative learning mechanisms be used within the model to generate stable state representations in the neuromorphic system to potentially improve ratiometric discrimination as a whole; and separately, can such neuromorphic processing of chemosensory signals achieve rapid and robust ratiometric decoding of real-world chemosensor input.

## Materials and methods

We adopt a multilevel modeling approach using both rate- and spiking-based neuronal models to first understand the broad properties of the MGC network before investigating the effect of learning based upon spike-timing and separately the capability of the implemented neuromorphic system for real-world chemosensor processing.

### Macroglomerular complex antennal lobe computational model

**Figure 1:**
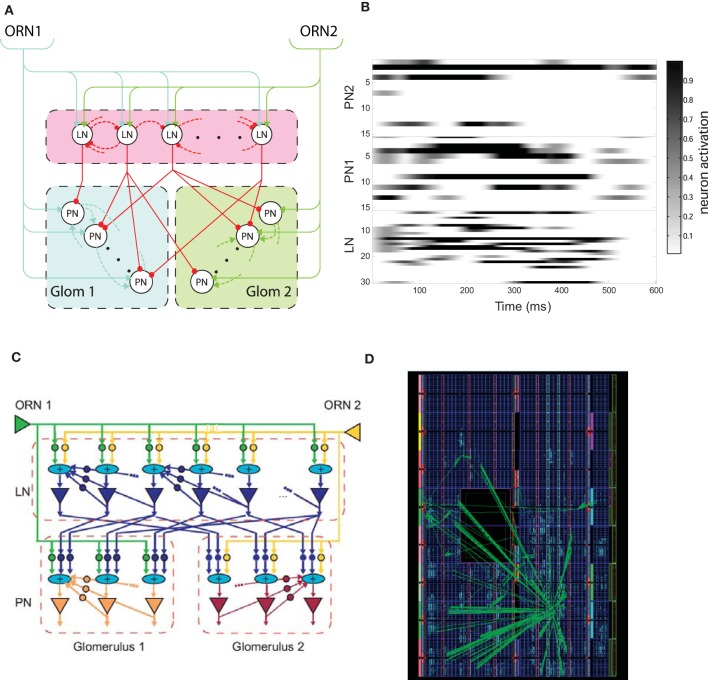
**(A)** Schematic of the insect antennal lobe macro-glomerular complex (MGC) comprising two types of olfactory receptor neurons (ORN) innervating the projection neurons (PNs) of their respective glomerulus (GLOM1 or GLOM2), and the population of local interneurons (LNs)—arrows and circles represent excitatory and inhibitory synapses, respectively. **(B)** Example neuronal dynamics for the LN and PN population in each glomerulus before learning, showing LCA dynamics (PN1 and PN2 subpopulations correspond to those PNs innervating GLOM1 and GLOM2 glomeruli, respectively) normalized to the maximum activation in response to a square pulse ORN stimulus of duration 500 ms beginning at time 0. The gray level shows the normalized activation level for each neuron. **(C)** Schematic of the neuromorphic MGC model showing the programmable logic neuronal model components and their interconnectivity—triangles correspond to leaky integrate-and-fire (LIF) neuron models, circles to exponential decay synapses and elliptical “+” elements correspond to current synaptic integration nodes. **(D)** Field programmable gate array (FPGA) floorplan of the physical device showing the footprint for the MGC model optimized down to 3095 of the 11,200 available logic slices of a Xilinx Virtex 5 device [**B** and **D** reprinted with permission from Rácz et al. (2013)].

To perform ratiometric processing of real-time chemosensor input we first developed a biologically-constrained computational model of the early stages of ratiometric processing in insects, the MGC of the AL, in Matlab (delete first) (Mathworks, USA) using probabilistic connectivity patterns based on morphological studies of the moth brain (Akers and O'Connell, 1991; Hansson et al., 1992; Sun et al., 1997; Hansson and Anton, 2000; Carlsson and Hansson, 2002; Carlsson et al., 2002)—shown in schematic form in Figure 1A and described in detail in (Chong et al., 2012).

Briefly, two types of ORN (ORN 1 and ORN 2, Figure 1A) drive excitation within the model, being connected to LNs and PNs through excitatory synapses (shown as arrows). The model consists of two glomeruli where every LN receives input from both ORNs but the PNs of each glomerulus are activated only by a specific ORN type. LNs have a probability to form inhibitory interconnections between themselves (shown as dashed red connections) and PNs belonging to the same glomerulus have excitatory interconnections (dashed blue and green connections) and provide the main output of the MGC to higher centers of the insect brain. LNs can also make inhibitory connections with PNs of any glomerulus (red connections), again according to a probabilistic connectivity rule. For simplicity, feedback from PNs to LNs was not included in the field programmable gate array (FPGA) implementation presented in this article, since for the parameter ranges studied these additional connections made no qualitative change to the results.

Individual PN and LN neurons were first modeled using a first-order ordinary differential equation (ODE) to describe the evolution of the firing-rate over time, with independent and identically distributed noise added at each time-step sampled from a Gaussian distribution. Probabilistic connection rules between neurons were used to ensure that results did not depend upon specific weight or connectivity configurations—see Chong et al. (2012) for further continuous model details. Figure 1B shows the complex time dynamics in LN and PN population activity obtained in response to a fixed ratiometric input.

### Spiking neuromorphic implementation of MGC AL model in programmable logic

Our MGC AL model was then implemented in programmable logic in the form of a FPGA using an existing construction kit of programmable logic neuronal modeling components, comprising leaky integrate-and-fire neurons and dynamical synapses adapted by spike timing-dependent plasticity (STDP) (Guerrero-Rivera et al., 2006). The neuronal modeling construction kit exploits a fine-grained parallelism architecture, achieving iteration speeds well beyond biological timescales, but can also be clocked over a range of frequencies to match dynamics between the neuronal model and peripheral chemosensor input [see Experiment Proceducre in Materials and Methods].

Neuron somata were modeled as leaky integrate-and-fire (LIF) units such that below some threshold, *V*_θ_, the dynamics of the membrane potential, *V*(*t*), are described in the normal way by a first-order ODE of the form

(1) $$\frac{dV\left(t\right)}{dt}=-\frac{V\left(t\right)}{R_{m}C_{m}}+\frac{I\left(t\right)}{C_{m}},$$

where *R*_*m*_ and *C*_*m*_ are, respectively, the cellular membrane resistance and capacitance, and *I*(*t*) is the total dendritic input current arriving at the neuron (see below). The membrane potential at rest [when *I*(*t*) = 0 for all *t*] is conventionally set to 0, as per Equation 1, and the membrane time-constant is defined as τ_*m*_ = *R*_*m*_*C*_*m*_ (10 ms for ORNs and PNs, and at 20 ms for LNs). The terms contributing to *I*(*t*) are due to chemosensor responses (see Equation 8) if the neuron is an ORN, and to ORNs and lateral interactions if the soma belongs to a PN or an LN, according to the connectivity defined in the model (Figure 1A). Whenever the membrane potential *V*(*t*) reaches the threshold value *V*_θ_, the LIF is immediately reset to the after hyperpolarization value, *V*_ahp_, (that we took to be 0) and a spike is generated at the output of the soma.

To deploy the LIF neuron in FPGA hardware as a minimal complexity soma model using the minimum number of logic slices, we applied forward-Euler (FE) method of integration in discrete time steps (each of time period Δ), such that the membrane voltage for time step *k* + 1 is computed from

(2) $$V_{k+1}=V_{k}\left(1-\frac{△}{\tau_{m}}\right)+\frac{△}{C_{m}}I_{k},$$

which describes an exponential decay in the membrane potential (first term) superimposed upon an integration of dendritic current (second term) for each time step. The result of applying FE in this way is a soma unit that requires no floating arithmetic unit, but instead deploys two signed adders, one multiplexer, one shift register, one register, one comparator, all with arbitrary bit-length depending upon the required arithmetic precision (we used 32-bit word length throughout) and a one-bit one-shot monostable. We showed previously that because the FE scheme is applied here to the integration of linear ODEs exclusively, as being the case for all neuronal elements of the neuromorphic kit, our circuits produce an exact integration (EI) that is equivalent to the exact solution, simply by making a small correction to the true time constant, τ_*m*_, according to (Guerrero-Rivera et al., 2006)

(3) $$\tau_{m}\to \frac{△}{1-e^{-△/\tau_{m}}}\cdot$$

This fact guarantees that the numerical error for any model constructed from components from our neuromorphic construction kit is randomly distributed within ± ½ least significant bit to produce no systematic propagating error overall.

Dendritic current resulting from presynaptic spikes (both excitatory/inhibitory postsynaptic potentials (EPSC/IPSCs)) arriving at times (*t*_1_, *t*_2_, … *t*_*e*_, … *t*_l_) within the MGC AL model are described using exponential decays, to give the total dendritic current driving each soma as

(4) $$I\left(t\right)=bw_{i,j}\sum_{e=1}^{l}H\left(t-t_{e}\right)e^{-\frac{t-t_{e}}{\tau_{e}}},$$

where *H*(•) is the Heaviside function and *b* is some constant, τ_*e*_ is the time constant of the exponential decay of the resulting EPSC for each arrival spike (or τ_*i*_ for the corresponding IPSC) and *w*_*i,j*_ is the synaptic efficacy (weight) for connections between neuron *i* and *j*. Similarly to the approach taken to the LIF soma, Equation 4 can be approximated using the FE scheme to calculate the total dendritic current at time step *k* + 1, according to

(5) $$I_{k+1}=bw_{i,j}\delta_{k+1,e}+I_{k}\left(1-\frac{△}{\tau_{e}}\right),$$

where δ is the usual Kronecker delta such that a current pulse of magnitude *bw*_*i,j*_ is injected during a time step containing the presynaptic spike arriving at time *t*_*e*_ (first term), which is superimposed on an exponential decay in the current for each time step (second term). As with the LIF implementation, correcting the time constant for the synapse, τ_*e*_, according to Equation 3 again guarantees an EI solution, such that there is no systematic propagating numerical error. The resulting EI synapse circuit implemented in FPGA requires two signed adders, one multiplexer, one shift register and one register, again with arbitrary bit-length depending upon the required numerical accuracy. Register transfer level designs for all programmable logic neuronal modeling kit components are provided in (Guerrero-Rivera et al., 2006).

The relationship of the modeling kit components and MGC AL model is shown in Figure 1C (LIF soma depicted as triangles and inhibitory/excitatory synapses as circles). Recurrent feedback in the model that underlies the complex spatiotemporal dynamics shown in Figure 1B is due to both LN and PNs outputs being reciprocally connected to their equivalent cell type inputs. Current summation nodes receiving input from multiple converging synapses (shown as “+” symbols) simply sum multiple EPSC and IPSCs generated at each time step. The complete AL MGC model consisted of 2 ORNs, 30 LNs and 2 glomeruli, each containing 15 PNs with reciprocal synapses deployed according to the probabilistic connectivity rules.

Neuronal modeling kit component register-transfer level designs were coded in VHSIC Hardware Description Language (VHDL) and logic synthesis was completed using ISE Design Suite (Xilinx Inc., USA) and Precision Physical (Mentor Graphics, USA) for the target FPGA device (Xilinx Virtex®-5 FX70T). The resulting design is massively parallel in that model kit components share no common computational resources and each computation within each component complete after a single clock cycle, resulting in one model iteration step per clock cycle overall. Thus, the *ca.* 2 ns clock cycle period of the Xilinx device (550 MHz clock frequency) renders hyper real-time simulation of the neuronal model at least 3 orders of magnitude faster than biological timescales, that could potentially be traded for model scale through time multiplexing should scalability in the model become important. The clock frequency of the MGC AL model core within the FPGA can be adjusted accordingly to match the dynamics of the peripheral chemosensor input using on-chip digital clock managers. The resulting floorplan of the MGC AL design is shown in Figure 1D and occupies 3095 of the available 11,200 logic slices available on the device.

To permit comparison between the continuous version of the model and its spiking neuromorphic variant, as well as for use in the ratiometric classification (see below), we convolved spikes produced by the PN LIF somata with a discrete Gaussian kernel to generate a real-time estimate of the firing rate, *r*_*k*_, at each time step, *k*, from a generated pattern of spikes at times (*t*_1_, *t*_2_,… *t*_*e*_,… *t*_*l*_), analogous to the activation level in the continuous version of the model, as follows

(6) $$r_{k}=\sum_{n=0}^{k}\delta_{k,e}T\left(n,d\right)\text{​},$$

where *T*(*n, d*) = *e^−d^I_n_(d)* is the discrete Gaussian kernel summed over each spike occurrence estimated from a modified Bessel function of integer order, *I*_*n*_(*d*), implemented as a lookup table in the FPGA and *d* determines the kernel window width that was selected to correspond to the time constant of the cell, τ_*m*_. We checked that the dynamics of the resulting PN and LN firing rates computed from the discrete spiking LIF version of the model using Equation 6 qualitatively matched those displayed in the continuous version of the model. Both discrete and continuous versions of the MGC AL model displayed comparable behaviors with identical operating parameters and input excitation ratios.

### Spike timing-dependent plasticity

Although associative learning mechanisms have not yet been directly shown specifically for MGC LNs and PNs in the AL of insects, more widely in the insect AL adaptation from associative conditioning of specific ratios of chemical components has been demonstrated requiring plasticity (Fernandez et al., 2009). Moreover Hebbian-like STDP has been recently demonstrated for downstream readouts of AL PN information in Kenyon cells (Cassenaer and Laurent, 2012). For this learning mechanism we implemented the widely accepted STDP (Abbott and Nelson, 2000; Guetig et al., 2003; Caporale and Dan, 2008) for ORN-LN and LN-LN connection weights to adapt the corresponding exponential decay synapses. This provided a convenient unsupervised learning method to test the ability of our neuromorphic model to robustly discriminate chemosensor input in real-time by learning blend ratios.

Depending upon the difference in pre- and post-soma spike timing, Δt, for each LN soma, the weight was adapted at each iteration step according to the asymmetric STDP rule

(7) $$△w_{i,j}\left(△t\right)=\{\begin{array}{ll} △W^{-}\left(1-\frac{\tau_{-}}{\tau_{-}+\tau_{+}}\right)e^{-\frac{△t}{\tau_{-}}} & \text{for}\Delta t>0\\ △W^{+}\frac{\tau_{+}}{\tau_{-}+\tau_{+}}e^{-\frac{△t}{\tau_{+}}} & \text{for}△t\leq 0 \end{array},$$

where Δ*W*^−^ and Δ*W*^+^ are the weight adaptations and τ_−_ and τ_+_ time constants (each set to 20 ms) for long-term depression (LTD) and long-term pontentiation (LTP), respectively, that governs the weight decrement or increment for each pre-/post-soma spike pair that vary dependent on the biological context. Thus, if the presynaptic cells (in this case ORN or LN) produces a spike before the target cell (in this case LN) the weight increments, otherwise it decrements. This arrangement potentiates those synapses that generate EPSCs capable of driving the soma beyond threshold to fire. The exponential shape of the LTD/LTP kernel means that spike pairs closely associated in time create strong potentiation and depression in the synapse.

The register-transfer level design used in our FPGA implementation for the STDP adapting mechanism is shown in (Guerrero-Rivera et al., 2006). Since signed 16-bit integer representation was used to store each weight value, −32,767 to +32,768 provide the minimum and maximum weight values in hardware. We found that weight adaptations close to Δ*W*^−^ = Δ*W*^+^ = 150 quickly produced stable weight values (see Results). We started from initial LN and PN weights values drawn from random uniform distributions ranging from +1,000 to +3,000 for ORN to LN, and from +100 to +400 for LN to LN connections, although neither of these conditions strongly altered the asymptotic values in the model weight configuration after adaptation.

### Experimental procedure

**Figure 2:**
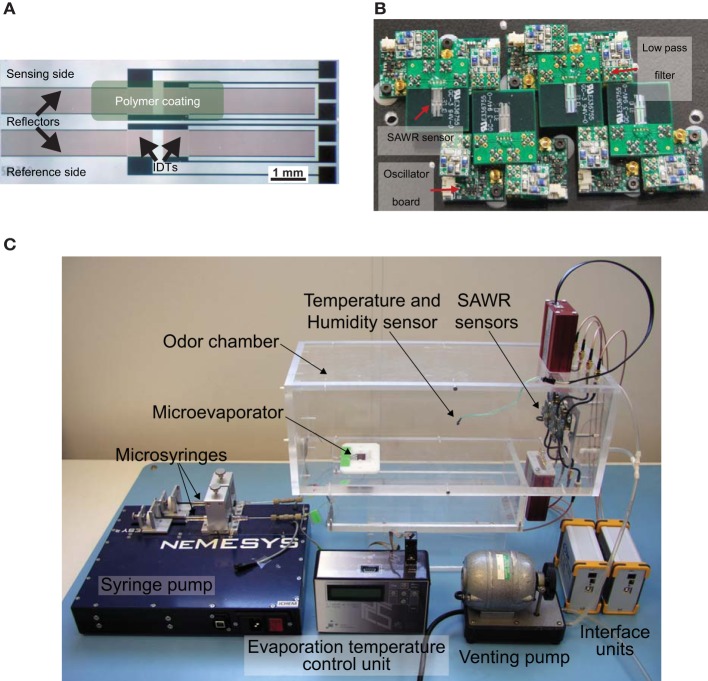
**(A)** Optical micrograph of a 262 MHz Rayleigh mode dual surface acoustic wave resonator (SAWR) chemosensor fabricated on X−propagating ST−cut quartz substrate with aluminium electrodes for air phase measurements. Each SAW resonator comprises a coated and non−coated reference device for drift and temperature compensation (see text). **(B)** Photograph of a 4-chemosensor SAW array together with the associated radio frequency oscillator and RF amplifier circuits to drive the acoustic resonator and low pass filters. **(C)** Experimental set-up for SAWR chemosensor array data-set [reprinted with permission from Yang et al. (2012)]. Specific ratios of volatiles are delivered into the odor chamber via the dual-line programmable syringe pump driving a micro-evaporator to generate a binary mixture headspace in a precise mixture. SAWR chemosensor array and associated electronics are controlled by a PC interface and SAWR outputs driving the neuromorphic FPGA processor shown in Figure 3.

The real-time neuromorphic MGC AL model input was the time-dependent frequency signals *f*(*t*) obtained from a surface acoustic wave (SAWR) chemical microsensors shown in Figure 2A. The microsensors were designed in a dual configuration, each operating at a baseline resonant frequency of 262 MHz. The acoustic wavelength of the device is 12 μm, having been defined by the interdigitated transducer (IDT) geometry and piezoelectric properties. The Rayleigh SAWR was fabricated with aluminum IDTs and reflectors by a lift-off technique on an ST-cut quartz substrate by SAW Components GmbH (Dresden, Germany). ST-cut quartz substrate was selected because it provides excellent temperature stability near room temperature (Sang-Hun et al., 2005).

An experimental procedure was designed to expose the SAWR devices to precisely controlled binary blends of two naturally occurring fruit volatiles: namely 3-methylbutan-1-ol and ethyl acetate. One half of each dual sensor was coated with a non-conducting polymer film specifically selected to detect the target compound, whilst the other half was left uncoated to serve as a reference channel. Hence the differential signal from the dual device has reduced ambient interference/noise and better output stability. Commercially available polycaprolactone (PCL) and polyvinylcarbazole (PVK) stationary phase polymer materials were used as the chemical coatings to detect 3-methylbutan-1-ol and ethyl acetate volatile vapors due to their high sensitivity and reversible responses. The polymer compounds were dissolved in toluene to make a 0.25%wt mixture on a stirring hotplate (SEA MS-506, Scientific & Educational Aids Ltd., Windsor, United Kingdom) for 12 h, then sprayed onto the sensor surface with a commercial airbrush (Iwata HP-BC1P, Anest Iwata Corp., Yokohama, Japan). The polymer coating thicknesses were estimated based on the measured frequency changes (145 kHz for PCL and 111 kHz for PVK) to be only 15 nm for PCL and 11 nm for PVK using the calculation method described in (Sang-Hun et al., 2005). Further details on the SAWR chemical sensors are provided in (Rácz et al., 2013; Yang et al., 2012).

Table 1 gives both the liquid volumes of the compounds released and the five precise volumetric ratios^1^. Ratios were injected using a programmable syringe pump (Cetoni GmbH, Korbussen, Germany) and pre-mixed in a micro-reservoir (0.375 μ l) into a sampling chamber housing the SAWR chemosensors ensuring complete evaporation through a temperature-controlled (to 105°C) micromachined evaporator based upon endocrine gland of an insect (Bula et al., 2012). Figure 2C shows the experimental set-up. A venting pump was used to terminate rapidly the chemical signal at the end of the stimulus period and to remove any chemical residue between trials. The data-set was collected using a randomized sequence of ratios to minimize any systematic error due to polymer layer or other time-dependent effects.

**Table 1:** **Ratio component concentrations used in the SAWR chemosensor experiments**.

**Ratio number and volumes**	**3-methylbutan- 1-ol (μl)**	**Ethyl acetate (μl)**
R1—1:4	4	16
R2—1:2	6.7	13.3
R3—1:1	10	10
R4—2:1	13.3	6.7
R5—4:1	16	4

### Neuromorphic programmable logic processor integration

**Figure 3:**
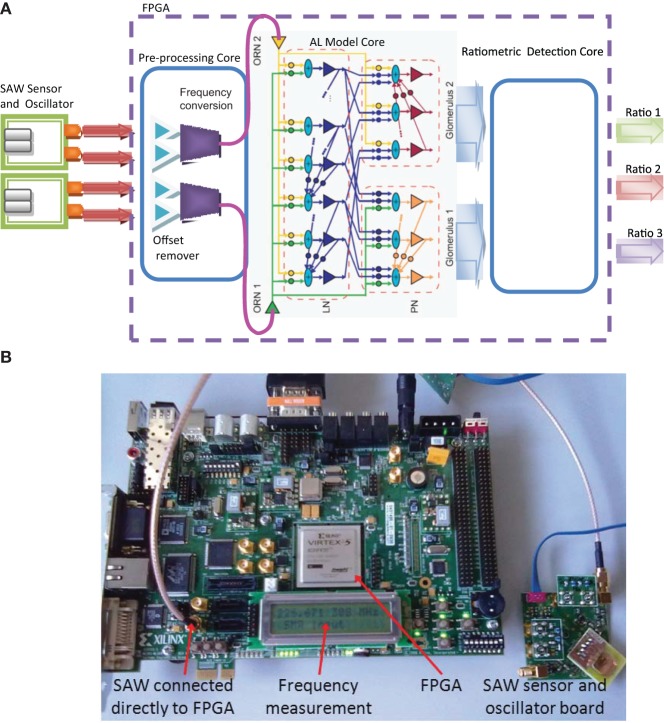
**(A)** Neuromorphic MGC processor and its chemosensor array integration. The resonant oscillation frequency of the SAWR devices are measured in real−time by the FPGA through the pre−processing core that removes the common mode between reference and polymer−coated input signal channels (offset remover) and scales the chemosensor input signals linearly to drive the AL Model Core running in real-time. A ratiometric detection core receives the spiking PN population outputs from each glomerulus and classifies between set ratios according to a k-nearest neighbor trajectory rule (see Results). **(B)** Xilinx Virtex®-5 FX70T development board integrated with SAW resonator and associated electronics displaying the current SAW input frequency.

An FPGA logic development board based around the Xilinx Virtex 5 FPGA device was used as the target platform for the MGC neuromorphic processor (Virtex®-5 FX70T development kit, Xilinx, Figure 3B) for interfacing with the SAWR devices. The Virtex 5 device contained three cores developed for the real-time processing of chemosensor data (Figure 3A). The first core subtracts the common mode (differential) signal between a pair of identical SAWR sensors, one of which was coated with polymer material and one not, so as to reduce systematic drift in the sensor signals. Thus, the differential signal driving each ORN was derived from a pair of chemosensors (coated and uncoated generated signals) by subtracting the integrated oscillation signals obtained from the coated SAWR device as a frequency, *f*_coated_(*t*), from that of the uncoated device, *f*_uncoated_(*t*), to generate the synaptic current driving the ORN leaky LIF soma,

(8) $$I\left(t\right)=\beta (f_{\text{coated}}(t)-f_{\text{uncoated}}(t)).$$

Since there is no spontaneous firing in the model, we ensured that the dynamic range of the sensor was appropriate by choosing the constant β such that the maximum frequency change in the sensor across the data-set corresponded to a maximum ORN firing rate of *ca.* 50 Hz., close to that reported for ORNs *in vivo* (Rospars et al., 2000). The resultant two sensor pair signals were provided as an input to the neuromorphic model core—the AL MGC model, consisting of 2 ORNs, 30 LNs and 2 glomeruli each containing 15 PNs. The output of the AL model (30 PNs) was used as an input to the third and final stage of the chemoreceiver, the ratiometric detection core. The core compared the MGC PN model output trajectories for an unknown ratio input with those stored internally corresponding to known classes using a *k*-nearest neighbor classifier.

### Ratiometric detection core

We applied a simple *k*-nearest neighbor algorithm using Euclidean (linear) distance to classify the blend ratio and applied this equivalently to the normalized chemosensor time series trajectories and MGC model PN output trajectories for the available repeat trials. First we calculated an average firing rate trajectory across multiple trials for the entire PN population from the continuous version of Equation 6, as a function of time for each ratio class, ${\vec{r}}^{x}(t)$, (ratio class *x*) from both the PN population activity for each trial *y*, such that ${\vec{r}}^{x,y}\left(t\right)=\left(r_{1}\left(t\right),r_{2}\left(t\right),\;\ldots \;r_{n}\left(t\right)\right)$ for all PNs in the MGC model and separately for the normalized chemosensor time series at the ORN inputs from Equation 8, ${\vec{r}}^{x,y}(t)=(I_{1}(t),I_{2}(t)),$as follows

(9) $${\vec{r}}^{x}(t)=\frac{1}{n}\sum_{y\;\in \;x}^{n}{\vec{r}}^{x,\;y}(t),$$

using the available repeat trials for each class *y* ∈ *x*, where *n* is the total number of trials in each class. *k*-nearest neighbor then proceeds by computing the nearest mean class trajectory, ${\vec{r}}^{x}(t)$, to a new unseen trajectory ${\vec{r}}^{\text{unseen}}(t)$by averaging the Euclidean distance between the two trajectories over their length, up to the decision time, *T*

(10) $$D_{x}=\frac{1}{T}\int_{0}^{T}dt\left\|{\vec{r}}^{\text{unseen}}(t)-{\vec{r}}^{x}(t)\right\|.$$

We then choose class *k*, such that *D*_*x*_ is minimized (*k*-nearest neighbor). Repeating this procedure for both the trajectories of MGC model PN output population in response to the normalized SAWR data and the normalized chemosensor SAWR trajectories themselves, allowed us to assess if the MGC model could classify the ratio input sequences more accurately and in faster time, *T* (see Results).

Additional control logic and memory was required to operate the FPGA design. Model configuration parameters are stored in the FPGA block memory (BRAM) and then distributed to their corresponding synapse by means of a decoder, enabling a register to store the configuring parameter. Once all parameters are stored in their corresponding register, the MGC neuronal model core operates with the state outputs stored into two BRAM memories, for PN and LNs. The internal model states were simultaneously sent to the host PC and stored for offline processing and analysis.

The MGC FPGA model was tested by generating ORN input through two function generators, each generating a square pulse of a frequency varying between 0 and 10 kHz, to test the model under a variety of input conditions. The output of the PN neuron population was then compared to a Matlab implementation, through custom software to interface the hardware VHDL modeling environment (Xilinx ISE DK and Mentor Garphic ModelSim simulator) to generate ORN input signal to feed both the Matlab and FPGA models, and then read the FPGA output in Matlab for analysis and direct comparison of the resulting behaviors. Both FPGA and MATLAB implementation gave identical numerical results within ± ½ least significant bit.

## Results

We first present results obtained from the continuous variant of the MGC model with non-adapting synaptic weights in order to assess the ratiometric dependence of the dynamics in two operating regimes. We next show results for an equivalent discrete-time spiking implementation of the MGC model with adapting synaptic weights in order to assess if STDP learning can generate stable weight configurations to improve ratiometric discrimination. Finally, we drive the spiking model implemented in programmable logic with non-adapting synapses optimized using a “greedy” algorithm for processing transient chemosensor signals in real-time and assess the classification accuracy of chemical blend ratios.

### MGC antennal lobe model produces limit-cycle and fixed-point ratio dependent trajectories

**Figure 4:**
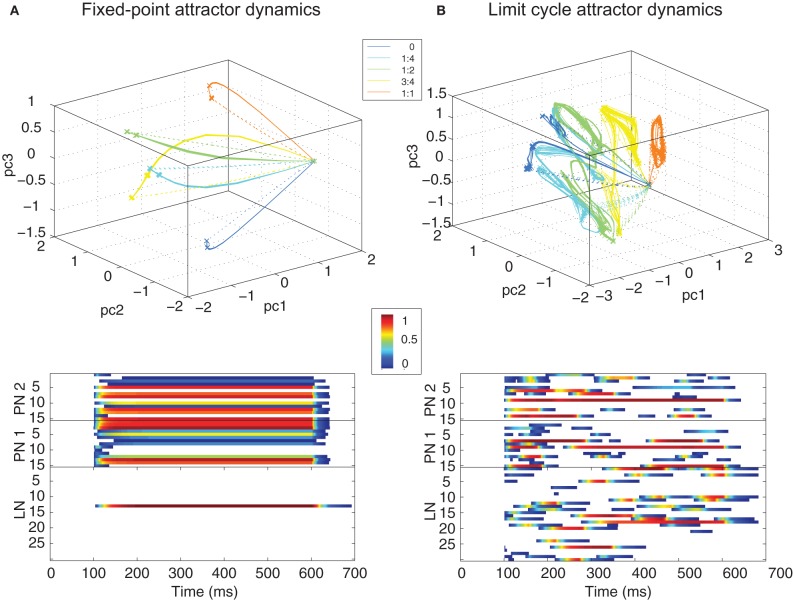
**Trajectories (top panels show 5 different ratios) and raster plots (bottom panels show ratio 1:2) of the PN population activity for different classes of dynamics observed in the MGC AL model: (A)** fixed–point attractor (FPA), and **(B)** limit cycle attractor dynamics (LCA). Solid and dashed lines in the trajectories show transients during stimulus onset and offset respectively. The stimulus lasted for 500 ms, starting at 100 ms and ending at 600 ms (color scale in the rasters represents normalized firing rate).

Depending upon the connectivity probabilities in the LN population of the model, the model displays two categories of behavior—fixed points attractor (FPA) and limit cycle attractor (LCA) dynamics (Figure 4). LCA dynamics, which was observed when LN cells were mutually connected asymmetrically through the probabilistic connection rule (*p*_connection_ = 0.25), is characterized by a switching behavior that imposes slow temporal patterning in activity and rich spatiotemporal dynamics. FPA dynamics on the other hand, were observed when mutual LN connectivity was all-to-all (*p*_connection_ = 1 and thus symmetric).

Without adaptations in synaptic strength and depending upon the probability of connection between LNs, the PN population displayed both FPA and LCA dynamics when stimulated with a synthetic square ORN input pulse of different ratios (Figures 4A,B, respectively). Trajectories of the PN responses to specific ratiometric inputs were represented using PCA applied to the firing rate outputs of the model. FPA dynamics were characterized by a rapid change in the PN population output to a fixed-point attractor in the phase space that was stable for as long as the stimulus was maintained. Since no spontaneous firing was used in the model, the PN population quickly returned to zero activity after stimulus offset (dashed lines in the trajectory figures). LCA dynamics, in contrast, caused PN population trajectories to oscillate about an attractor for as long as the stimulus was present. Importantly, for both LCA and FPA modes of behavior, the MGC network produced population dynamics that were found to be specific to the ratio of activity in the ORN inputs in a binary blend. In each case the attractor for PN population trajectories is found to be specific to the ORN1:ORN2 ratio of input activation and varies smoothly dependent upon that ratio.

The normalized firing rate activities over time (bottom panels of Figure 4 show ratio 1:2) for both PN and LN populations show very different behaviors in each dynamical regime—FPA results in a single “winner-takes-all” competition in the LN population, whereas LCA results in a so-called “winnerless competition” exhibiting relatively slow spatiotemporal patterning in the PN and LN population activity with no neurons maintaining dominance for a sustained period.

Moreover, the attractor location was found to vary continuously within this phase space depending upon ratio. For FPA dynamics, the network encoded ratios through the spatial identity of neurons, whereas LCA networks produced a PN output continuously varying in time (bottom panels in Figure 4). While both operating regimes were able to encode the pheromone ratio reliably, we observed in previous work (Chong et al., 2012) that the ratio encoding was faster and more accurate using LCA dynamics (see Discussion). Timing simulations of the MGC design were conducted in ModelSim (Mentor Graphics, USA) and showed complex spatiotemporal dynamics in both PN and LN cell equivalent to those found in the continuous version of the model.

### MGC model behavior under spike timing-dependent plasticity (STDP)

**Figure 5:**
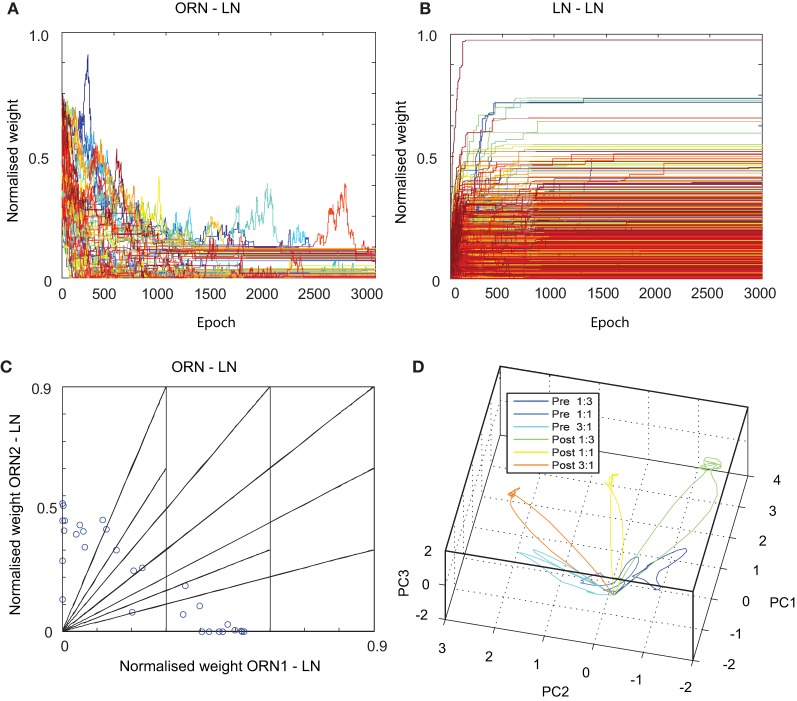
**Results of spike timing-dependent plasticity (STDP) applied to (A) ORN-LN and (B) LN-LN connections in the MGC AL neuronal model.** Adapted weights of both connection types are shown to converge from an initial randomized state to stable asymptotic values over many epochs. **(C)** Normalized synaptic weights of ORN-LN connections show that LN input tunings (blue circles) covers the space of possible input ratios (shown as linear segments) after STDP learning. **(D)** PN firing rate trajectories to three different ORN1:ORN2 input ratios (1:3, 1:1, and 3:1) both before and after STDP adaptation show greater attractor separation after learning (3 dimensional PCA explains 90% of total variance).

We were interested to understand whether an associative, Hebbian-like, plasticity rule could be applied to the model to create a stable configuration of weights (defined as a training epoch in which no weight changes occur in the model). For this we applied asymmetric STDP (see Materials and Methods) as a commonly adopted associative Hebbian learning rule variant for spiking models, that has been shown to account for a range of perceptual learning phenomena (see Discussion). We confined our STDP investigations to synaptic targets of the LN population, as being the key determinant for the ratio specificity in the model. As such, STDP synapses were used in the model for all excitatory ORN-LN and inhibitory LN-LN synapses (Figure 1). To evaluate the stability of STDP in the model we created a synthetic ORN input training data-set based upon a simple first-order chemosensor description. The training set consisted of a train of square pulses (each pulse lasting 100 ms, with 100 ms inter-pulse interval), filtered by an RC circuit (10 ms time constant), with amplitudes of each successive pulse defined as a concentration ratio ORN1:ORN2 with an arbitrarily defined and randomized order, 0:0, 0.5:0.5, 0.5:1, 0.5:1.5, 1:0.5, 1:1, 1:1.5, 1.5:0.5, 1.5:1, 1.5:1.5, with Gaussian noise added at each time step. The resulting pulse sequence was used to drive the ORN soma current, as *I*(*t*) in Equation 4 and repeated for 3000 epochs, over which the synaptic weights of the connections between ORN-LN and LN-LN converged to a distribution of steady state values (Figures 5A,B).

After training we investigated the ORN-LN weight configuration for each of the 30 LNs in the network (Figure 5C), that showed a stable configuration of weight pairs spread across the weight space of possible input ratios, deriving from competition between LNs to represent input ratios fed to the network. Thus, the result of STDP adaptation on ORN-LN and LN-LN synapses was that each LN was tuned to respond optimally to a different input ratio of input magnitudes defined in the training set and as a population they competed for coverage of ratio input space, mirroring a well-known result of reciprocally connected inhibitory networks using correlation based learning rules.

Finally we studied the effect of training on the PN population responses. After STDP adaptation in the model, we tested the identical set of ratio amplitudes, filtered by the same RC circuit and perturbed by the same noise as defined in the training set. For each ratio, the firing rates of the 30 PNs were reduced to 3-D trajectories using PCA (Figure 5D). The result is a clear amplification in the trajectories in response to the trained ratio categories, demonstrating a greater separation in the phase space during the pulse stimulus presentation. Interestingly, such increased separation in PN responses is reminiscent of that found in the AL of honeybees after odor-reward pair conditioning (Fernandez et al., 2009; see Discussion).

### Neuromorphic blend processing of early chemosensor transients

**Figure 6:**
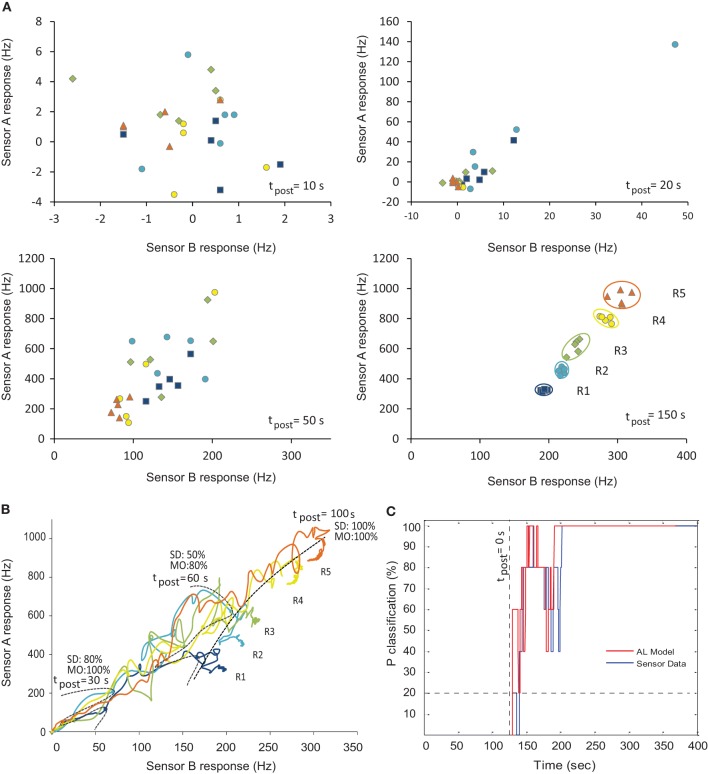
**Ratiometric classification performance of the neuromorphic MGC AL model over time. (A)** SAWR input chemosensor pair responses for different chemical ratios (see Table 1) at different points in time of the transient response post stimulus. **(B)** SAWR chemosensor pair raw data trajectories showing the difficulty of separation according to ratio during the transient phase (before 100 s post-stimulus). Time frontiers are shown at 30, 60, and 100 s post-stimulus, together with the classification probability (*P*_classification_) for the sensor data (SD) and model output (MO). **(C)** Real-time classification probability (*P*_classification_) of raw sensor data compared with MGC AL model outputs over time. During the early transient phase of the chemosensor input (*t*_post_ < 100 s post-stimulus), AL model output can be classified more robustly above chance compared with raw chemosensor input. As correct classification requires 1-of-5 categories, all with equal priors, chance level is 20% (shown by horizontal dashed line). R1, R2, … R5 represent distinct chemical ratios (see Table 1).

In order to assess the ability of our computational MGC AL model to decode blend ratios from realistic chemosensor input, we selected time series from two SAWR devices that showed as orthogonal (mutually exclusive) responses as possible to binary blends to represent the highly-specific input of receptors to the MGC area [PCL and PVK; see Materials and Methods]. Pre-synthesized fruit volatiles 3-methylbutan-1-ol (3 M) and ethyl acetate (EA) were presented in five different ratios (see Table 1). These time series were first normalized and then used to drive the two ORN inputs to the MGC model, according to Equation 4. Figure 6A shows the pair of SAWR chemosensor responses at different points in time post stimulus, whilst Figure 6B shows the trajectory of the SAWR chemosensor pair over time post stimulus. Separation of the raw data according to ratio identity is shown to be minimal before equilibrium in the responses, demonstrating the difficulty of the early transient ratio classification problem.

An important motivation of applying such neuromorphic processing to real-world chemosensor data is to assess whether signals may be classified more accurately using the MGC model compared against a standard classification technique applied to the raw chemosensor input. We wanted to know if we could classify the ratio being presented to the chemosensor array faster and with more accuracy using the MGC neuromorphic model. To address this we integrated SAWR chemosensor devices with differing polymer coatings with the FPGA based neuromorphic model, each displaying selectivity to two different fruit volatiles. The chemosensor interface circuitry is configured to drive the current in the ORN input and hence directly controls the firing activity of the model inputs according to Equations 4, 8. It was necessary to match both the sensor dynamic range (see Materials and Methods) as well as frequency response to those suited for the model. Driving the model with these chemosensor series generated an output trajectory of PN activity in 30 dimensions.

Random instantiations of the MGC models were generated using probabilistic weight and connectivity rules with STDP disabled and a greedy algorithm was used to select the optimal MGC model across the training set by classifying according to this method. Bootstrapping in the form of cross-validation was used to rotate the training and test data-sets to produce a probability of correct classification (*P*_classifaction_) over time. Figure 6C shows the comparison directly in the average classification performance at each time point, demonstrating faster ratio classification (the point at which classification rises above chance) performance using the MGC model output compared to chemosensor data directly, as well as greater or equal ratio classification across all time points.

## Discussion

We have described the implementation of a spiking MGC model of the insect AL in neuromorphic programmable hardware as a real-time solution to ratiometric processing of infochemicals, which builds upon two areas of existing work. Firstly, our existing firing-rate based model of the MGC AL (Figure 1A) was used in order to understand the fundamental network dynamics without learning (Chong et al., 2012). Secondly, we have made use of our existing spiking programmable logic neuronal modeling component kit (Guerrero-Rivera et al., 2006) to develop a spiking variant of this MGC AL model as a real-time neuromorphic hardware implementation based upon LIF neurons and STDP-adapting dynamical synapses (Figures 1C,D) that uses the same topology and architecture as the original model (Figure 1A). STDP deployed in a subset of the synapses in the spiking model as a local associative learning rule was shown to organize weights into a stable configuration after exposure to a randomized training set of synthetic data comprising a variety of input ratios. The result was greater separation in the PN phase space trajectories during the pulse stimulus presentation of trained ratio categories. Finally, the neuromorphic implementation without STDP enabled was tested for processing of chemosensor signals in real-time and shown to achieve robust ratiometric classification during the early transient phases of the response.

Existing computational models of the insect AL processing can be separated into two distinct categories: AL processing of general odors (Getz and Lutz, 1999; Bazhenov et al., 2001a,b; Martinez and Montejo, 2008; Capurro et al., 2012) that emphasize highly dimensional olfactory input required for general odor processing, through to computational models focusing on pheromone processing with relatively few stimulus dimensions (Linster et al., 1993, 1994; Av-Ron and Rospars, 1995; Av-Ron and Vibert, 1996; Linster and Dreyfus, 1996; Zavada et al., 2011; Chong et al., 2012). Early pheromone processing models by Linster and co-workers emphasized the role of oscillations arising between the interaction of LN and PN neurons. (Linster et al., 1994) describe a highly abstracted oscillatory model, showing that by balancing numbers of inhibitory and excitatory neurons in a network receiving two-component input, a system can be made to oscillate when a particular ratio is presented. (Linster and Dreyfus, 1996) describes a more biologically constrained model including LNs that mediate information to PNs. Again, the balance between inhibitory and excitatory elements is investigated, this time to show that PN response patterns can be made dependent on the input ratio. The model could be tuned such that PNs will display both inhibitory and excitatory influence from the LNs when a particular ratio is presented. As such, PNs respond with a pattern of activation, which includes mixed periods of excitation and inhibition, only for input that is close to a particular ratio.

Oscillations emphasized in these modeling approaches have been observed in many species such as locust, cockroach and some species of moth including *Manduca sexta* (Heinbockel et al., 1988), but not in other moth species, such as *Spodoptera litoralis* and *Agrotis ipsilon* (Zavada et al., 2011; Chong et al., 2012). This led (Zavada et al., 2011) to develop minimum complexity models of ratiometric processing in the MGC using exclusively feedforward competition-based network descriptions that do not depend upon olfactory oscillations to encode ORN ratiometric information. Our MGC model accounts for both limit cycle oscillations (LCA) (Chong et al., 2012), reminiscent of “winnerless competition” dynamics (Rabinovich et al., 2000), that show close relationships to those phase trajectories observed in AL neuronal recordings (Stopfer et al., 2003; Mazor and Laurent, 2005; Geffen et al., 2009) and non-oscillatory behaviors (FPA dynamics, Figure 4) reminiscent of “winner-takes-all” dynamics, depending upon the symmetry in connectivity patterns between the LN population.

In Figure 4, the intrinsic MGC network dynamics dominate since the input is held constant after an initial step change. While the external state (the input) is constant in time the internal state is shown to encode the ratio in a reproducible way in both FPA and LCA dynamical regimes. Therefore, we had a choice of which form of dynamics to deploy for real-world chemosensor signal processing. For this we chose LCA encoding strategies for two reasons: (a) we have shown previously these exhibit greater specificity in the dependence of the dynamics on the input ratio and more rapid encoding of the stimulus input (Chong et al., 2012) that may make them advantageous for ratiometric classification applications, and (b) in the same study LCA dynamics were shown to be more sensitive to external input transients under a pulsed stimulus regime, which is likely to be important in extracting transient chemosensor information during ratiometric classification. In the case of LCA dynamics we are computing without fixed point attractors (FPAs), but rather limit cycle behavior that depends on the input and current network state in a complex non-linear relationship. Eventually the limit cycles will dampen in LCA mode to a step input, but only after an extended time period (Chong et al., 2012), showing that (a) we are computing before attractors are reached, and (b) these dynamics depend upon the history of the stimulus over extended time periods.

The LCA dynamical behavior exhibited in our MGC model has a number of close relationships to “winnerless competition” (Rabinovich et al., 2008) and reservoir computing in the form of liquid-state (Maass et al., 2002) and echo-state machines (Jaeger and Haas, 2004), all of which emphasize the processing of sensory signals without the use of FPAs, but rather as continuous trajectories in the network dynamics that are sensitive to the input. These approaches and the MGC AL model in LCA mode potentially solve the complex problem of transient processing through a complex relationship between the current internal dynamical state of the network (arising from its intrinsic dynamical properties) and both the present and previous history of the input. Considered in this way, the complex dynamics within these networks form a type of temporal to spatial conversion (Buonomano and Maass, 2009) that has previously been shown to simplify the problem of recognizing chemosensor transients (Muezzinoglu et al., 2009).

We were interested to know if the LCA dynamical properties of the MGC model could be similarly advantageous for the processing of ratiometric information from chemosensor input in real-time. We assessed this by applying a *k*-nearest neighbor classifier to both the PN population output trajectories and normalized chemosensor responses integrated over time according to Equation 10 until some decision time, *T*. Exploiting LCA dynamics was found to improve the time where the classification rose above chance, producing a more rapid classification in ratio, allowing also a more accurate classification with *P*_classification_ for the MGC model outputs above that of the normalized chemosensor data across all time points. Such temporally sensitive neuromorphic processing shown by our MGC AL model could become extremely valuable for improved chemical sensing through stimulus-dependent spatiotemporal patterning imposed on the chemosensor array to enhance discrimination (Gardner et al., 2007; Sánchez-Montañés et al., 2008), and also as part of an infochemical communication system in which rapid and robust transient processing is paramount (Cole et al., 2009a,b; Rácz et al., 2011, 2013).

Regarding the implementation of the learning process, we chose asymmetric Hebbian STDP within the MGC AL model for synaptic targets onto LN neurons as a commonly adopted associative learning rule to self-organize its weight parameters (Caporale and Dan, 2008). STDP potentiates weights for which presynaptic spikes precede post-synaptic spikes, emphasizing causal relationship between presynaptic spikes creating postsynaptic activity in LN targets. In the case of multiple synaptic inputs onto single target soma operating under a balanced regime of inhibition and excitation (as in the LN layer of the MGC model) previous modeling studies have demonstrated that when Hebbian STDP is invoked synapses compete to drive the target cell output so that normalization of total weight in the postsynaptic cell is unnecessary (Song et al., 2000). In the reciprocally connected LN layer of our MGC model we find that the result of this local competition for LN firing results in a stable weight configuration (Figures 5A,B) that leads to an activity-dependent competition in LNs for different ratios of ORN input (Figure 5C). Thus, STDP is capable of self-organizing the LN layer to represent the space of input ratios presented during training that could potentially be useful for self-organizing chemosensor inputs with different real-world tunings (Albert et al., 2002; Gill and Pearce, 2003).

We found that applying STDP to both excitatory afferent ORN-LN and inhibitory LN-LN synapses led to a stable and asymmetric weight configuration (Figures 5A,B) driving competition in the LN layer to represent the input space of possible ratios (Figure 5C). This behavior can be understood by comparing to other studies on reciprocally connected neural models adapted by associative learning, equivalent to the LN layer in our model. Hebbian-like associative plasticity combined with suitable learning constraints can enforce competition between synapses in these reciprocally connected networks (Miller and MacKay, 1994), such that afferent inputs may then compete to drive the neuron response and self-organize to represent different input features, which has long been studied as a model of cortical development [reviewed by Swindale (1996), Miller (1996)]. Pure Hebbian rules in themselves however do not include a synaptic depression term, potentially causing weight values to explode in such models without suitable normalization (Gill and Pearce, 2003). Modern associative learning rules such as STDP, on the other hand, effectively solve this normalization issue by balancing synaptic depression and potentiation asymmetrically through spike timing (Guetig et al., 2003). Through balancing long-term potentiation and depression together with imposing a time asymmetry, STDP has been shown to regulate weights within a quiescent operating range, but still drive symmetry breaking of weights, leading to input feature selectivity in a variety of reciprocally connected networks without any additional requirement for synaptic normalization (Song et al., 2000; Song and Abbott, 2001). More recently STDP based models have been shown to be capable of modulating input selectivity in coupled populations of excitatory and inhibitory neurons in the olfactory (Finelli et al., 2008; Linster and Cleland, 2010) and mammalian visual pathways (Young et al., 2007; Srinivasa and Jiang, 2013).

We found the equivalent learning behavior in our study for the LN-layer adapted through STDP: that stability is achieved in both ORN-LN and LN-LN weights over time together with a wide variation of selectivity to ORN input covering the ratiometric stimulus space without the requirement for a normalization mechanism. In this way, the LN population responses were found to be increasingly decorrelated as a result of learning, shown to lead in greater separation in the network output phase space trajectories to different input ratios (Figure 5D). The fact that this LN layer representation leads to enhanced separation can be explained in terms of our previous analysis for the continuous version of the MGC model: that more diversity in the LN layer weights causes greater decorrelation in PN output population trajectories over time that was measured to provide greater ratio specificity and classification accuracy of ratios (Chong et al., 2012).

Since in the LCA dynamical regime of our MGC model the external input was found to influence LCA dynamics more strongly than in FPA (Chong et al., 2012), this regime provides a greater opportunity to learn transient dynamics through adapting synapses such as STDP, as used in the experiment for Figure 5. Indeed, (Buonomano, 2005) showed that in a certain class of recurrent network the use of STDP learning could form more stable dynamics. Our results show that the ratiometric dependence of the dynamics after STDP learning is amplified in the response, showing greater separation (Figure 5D). Interestingly, similar increases in separation in AL dynamics have been demonstrated in the honeybee AL resulting from paired conditioning of specific odor ratios and rewards (Fernandez et al., 2009). While STDP has not yet been demonstrated in the insect AL some form of local associative Hebbian-like plasticity mechanism is likely to account for such phenomena.

A number of computational olfactory models have been applied to offline processing of real-world chemosensor input (Pearce et al., 2001; Gill and Pearce, 2003; Gutiérrez-Gálvez and Gutiérrez Osuna, 2006; Perera et al., 2006; Raman et al., 2006; Muezzinoglu et al., 2008, 2009; Karout et al., 2011; Martinelli et al., 2011; Ayhan and Müştak, 2012; Yamani et al., 2012a,b) as reviewed by Marco and Gutierrez-Gálvez (2009) and more recently as a Frontiers in Neural Engineering Research Topic (Huerta and Nowotny, 2012). Some of these bio-inspired and biologically constrained models demonstrate clear advantages for chemosensor array processing to overcome the manifold challenges inherent to machine olfaction, beyond existing engineered signal processing solutions. Since these studies focus on offline processing of chemosensor input, they cannot strictly be considered neuromorphic solutions in terms of real-time hardware. Separately however, hardware neuromorphic systems have been reported in both programmable logic and custom VLSI, mostly based on mammalian olfactory processing (Guerrero-Rivera and Pearce, 2007; Koickal et al., 2007; Beyeler et al., 2010; Ayhan et al., 2012; Imam et al., 2012), but at this stage only a small number have been tested on real-world chemosensor input (Guerrero-Rivera et al., 2009; Karout et al., 2011; Hsieh and Tang, 2012). In contrast, the present paper demonstrates a speed advantage for rapid neuromorphic processing of real-world chemosensor input by exploiting chemosensor transient dynamics, but in an insect based olfactory system. All of these neuromorphic solutions have been tested with offline data-sets collected in a controlled laboratory environment. The future challenge for neuromorphic olfactory systems will be to come out of the laboratory and demonstrate robust gains in performance under realistic uncontrolled conditions, such as within odor plumes and advected environments.

We fitted the dynamics of the model by adjusting the global clock rate of the FPGA neuronal model core, a convenient option with non-volatile digital-based neuromorphic systems, in contrast to their analogue counterparts. This became particularly important in this context since the SAWR chemosensors displayed relatively slow transient responses over a 50–80 s timescale (Figure 6A). Although the dynamics of the model were necessarily slowed down to match the chemosensors it would be capable of processing dynamical ratiometric input much faster than biological time (for instance as generated from optical and nanosensors). This is a challenging aspect of fully analog VLSI where adjusting processing speeds through component time constants can be difficult over many orders of magnitude necessary for some machine olfaction neuromorphic solutions (Koickal et al., 2007).

Programmable logic neuromorphic approaches exploit the inherent parallelism in neural circuits and has become a popular alternative to building fully custom neuromorphic VLSI. Since each element of our neuromorphic design components share no common computational resources, the resulting design is massively parallel in that model kit and each computation within each component complete after a single clock cycle, resulting in one model iteration step per clock cycle overall. In practice, the programmable logic kit we have used in this neuromorphic design can achieve a computational capability far beyond that necessary for the relatively small scale MGC network considered in this study, particularly when considering the matching of the model dynamics with the response time of the SAWR chemosensors (Figure 6). A slightly larger mammalian olfactory bulb network implemented using the same FPGA modeling kit achieved a speed up factor of 2500 over a single core 3 GHz Intel processor with simulation results stored to memory rather than disk (Guerrero-Rivera et al., 2006). From our more recent MATLAB simulations of the MGC network, this could potentially be operated at sufficient speeds to process SAWR devices in real-time on a modern PC. Yet, the FPGA solution used here provides future scalability of the network for larger, more complex, ratiometric decoding problems, and the opportunity for processing much faster chemosensors, such as optical/nano chemosensors responding at nanosecond timescales, as well as miniaturization to low-power FPGA devices with smaller numbers of gates for bio-robotics and embedded applications.

Since real-time large-scale measurement from biological olfactory receptors is almost within reach (Goldsmith et al., 2011) neuromorphic architectures, such as the programmable logic olfactory bulb (Guerrero-Rivera et al., 2006) running at biological timescales should soon offer the possibility to be connected directly to biological receptors and possibly even as the basis for prosthetics supporting early olfactory processing, communicating sensory information to the higher centres of the brain.

### Conflict of interest statement

The authors declare that the research was conducted in the absence of any commercial or financial relationships that could be construed as a potential conflict of interest.

## Footnotes

^1^Note that although the precise compound ratio of the liquid will not be the same as in air, nor indeed in the polymer coating, due to the different molecular weights and unknown partition coefficients, the principle of detecting distinct ratios is conserved.
